# Development and Validation of the Readiness for End-of-Life Conversations (REOLC) Scale

**DOI:** 10.3389/fpsyg.2021.662654

**Published:** 2021-03-19

**Authors:** Pia Berlin, Nico Leppin, Katharina Nagelschmidt, Carola Seifart, Winfried Rief, Pia von Blanckenburg

**Affiliations:** ^1^Clinical Psychology and Psychotherapy, Department of Psychology, Philipps University of Marburg, Marburg, Germany; ^2^Department of Medicine, Research Group Clinical Ethics, Philipps University of Marburg, Marburg, Germany

**Keywords:** end-of-life, psycho-oncology, cancer, communication, readiness

## Abstract

**Background:** Engaging in end-of-life care considerations is beneficial when the time is right. The purpose of this study is to provide a valid instrument to assess peoples readiness for end-of-life conversations before they are initiated.

**Materials and Methods:** A community sample was recruited in study one for exploratory factor analysis of a 13-item questionnaire. In study two, psychometric properties were analyzed with structural equation modeling in a population affected by cancer. Convergent and discriminant validity were assessed with questionnaires measuring distress, depression, anxiety, fear of progression, and distress of death and dying.

**Results:** In study one (*N* = 349) exploratory factor analysis resulted in three subscales readiness (α = 0.84), communication (α = 0.76), and values (α = 0.56) with a possible common factor (α = 0.84) for a community sample. In study two (*N* = 84) the three-factor solution with 13 items was not supported for cancer patients. Factor structure was adapted to 12 items with one common factor readiness (α = 0.87). Model fit was good: χ^2^(50) = 59.18, *p*>0.05 (Satorra-Bentler-correction = 1.27), with χ^2^/*df* = 1.184, *rRMSEA* = 0.053 (90%-*CI*[0.000;0.100]), and *rSRMR* = 0.072. Convergent validity was supported by moderate correlations to trait gratitude, ratings of readiness to provide a living will or talk with family about the end of life. Divergent validity was supported by no or small correlation with distress, depression, general and death anxiety and fear of progression, respectively.

**Conclusions:** Results support usage of the REOLC Scale in different settings with adapted factor structure. The questionnaire is interpreted as valid and reliable instrument to assess objective readiness for end-of-life conversations.

## 1. Introduction

Independent of health, death, and dying are inevitable parts of life, but preparation for this last challenge is often poor. When asked directly, people support the importance of end-of-life planning (Perkins et al., 2002; Lambert South and Elton, 2017; Banner et al., 2019). Preparations entail order of financial affairs, living painlessly, and maintaining dignity at the end of life (Steinhauser et al., 2011; Banner et al., 2019). Especially in medical context, participants seek opportunities to set boundaries to interventions and treatment options (Seymour et al., 2004; Steinhauser et al., 2011), minimizing stress and complications for and conflicts between family members (Seymour et al., 2004; Banner et al., 2019). Early end-of-life conversations (e.g., about values, fears, wishes, and preferences at the end of life) empower people to be fully informed and included in decision making processes (e.g., advance care directives) (Abba et al., 2013; Epstein et al., 2017) and have been proven to be beneficial. Wishes are more likely to be honored, quality of life, satisfaction and illness adaptation increase, hospice care is received earlier and more frequently (Detering et al., 2010; Wright et al., 2010; Abba et al., 2013; Bischoff et al., 2013; Brown et al., 2017). Relatives suffer less from depressive symptoms, post-traumatic stress, or anxiety during the final weeks and after bereavement (Detering et al., 2010; Abba et al., 2013; Banner et al., 2019). However, necessary end-of-life preparations are seldom undertaken before it is too late (Abba et al., 2013). Avoidance of preparations and low mortality communication may then result in unexpected financial costs and psychological burden on family members (Banner et al., 2019; Bachner et al., 2020). While patients often reported feelings of isolation, grief, anxiety or depression, caretakers may experience depression or complicated grief (Wright et al., 2010; Abba et al., 2013).

Participation in end-of-life conversations can be defined as health behavior (Fried et al., 2009). In order to understand motivations and intentions to improve and change health behavior, the Transtheoretical Model (TTM) proposes five stages (Prochaska et al., 2015). Precontemplation (no thought or intention to engage), contemplation (thinking about it), preparation (committing to behavior and preparing), action (engaging in health behavior), and maintenance (ongoing health behavior). In the dynamic process of health behavior change people can constantly move between stages and differ in readiness to engage and maintain them, so that challenges in healthcare include identification of ideal time points to initiate conversations about health behavior changes (Seymour et al., 2004; Lambert South and Elton, 2017). Key predictors that foster contemplation of end-of-life planning are having young children, decline in parents health, transition into health-care facility or loss of family members. Barriers to preparation and action for patients are fear of coercion, physicians who blindly follow advance care directives, lack of knowledge about treatment and prognosis or abuse by relatives (Seymour et al., 2004; Lambert South and Elton, 2017). Barriers for caregivers are avoidance of psychological burden and distress (Higginson and Costantini, 2002; Zhang and Siminoff, 2003; Goldsmith et al., 2007; Stone et al., 2012). To receive the best end-of-life care possible, interaction between patients and health care systems is needed without solely focusing on terminally ill patients (Gillick and Fried, 1995; Sinuff et al., 2015). Mortality communication barriers have been found to exist at all disease stages, but to intensify with prolonged illness (Bachner et al., 2020).

In addition to avoidance and unease of family members, one key element to overcome the cleft between wanting to engage in end-of-life discussions and fear to initiate them is trust in physicians. Trust is expected to develop and change in quality over time and contact, and to balance need for autonomy, care or additional need for information (Seymour et al., 2004). Still, it is common for patients to be expected to signal readiness for end-of-life conversations. Health care professionals are expected to successfully interpret signals based on intuition, sensitivity and common sense. However, without an objective assessment of readiness, up to 60% of patients were not approached to discuss end-of-life preparations although they were ready and underwent more aggressive treatment. Over the course of a disease, patients readiness and perceptiveness may change or never develop so that health care professionals need an objective measure assessing readiness more frequently (Maciejewski and Prigerson, 2013). Wrongful initiation of end-of-life conversations may result in emotional burden, lost hope and trust in physicians.

These findings highlight the importance of timing, context and of taking readiness for end-of-life conversations into account, before directly approaching patients (Abba et al., 2013; Simon et al., 2015). Solely relying on people to be terminally ill, physicians interpretation of readiness and trusting caregivers to know of preferences and wishes might lead to distress and rejection of end-of-life programs. Being unprepared or “not ready” could further lead to experiences of grief and death anxiety (McLeod-Sordjan, 2014). The purpose of this study is to develop and validate an evaluated method to assess readiness to engage in end-of-life conversations for a community sample and a population affected by cancer, respectively.

## 2. Study One

The first study (Clinical Trials: NCT03387436) compared three interventions to decrease experienced death anxiety distress and improve communication about the-end-of-life focusing an a community sample (von Blanckenburg et al., 2020). This paper only reports results relevant for questionnaire development and exploratory factor analysis.

### 2.1. Materials and Methods

#### 2.1.1. Inclusion Criteria and Procedure

Participants were eligible when aged 18 years or older, understood German sufficiently, did not suffer from dementia, suicidal thoughts (Beck et al., 1996), or acute psychosis (Wittchen et al., 1997) (Supplementary Material). Recruitment was completed using university email distribution, flyer and announcements or social media. Eligible participants were either provided with a paper-pencil or online form of the questionnaire and recruited in two rounds.

#### 2.1.2. Measures

##### 2.1.2.1. Readiness for End-of-Life Conversations Scale

A novel self-report scale was developed by experts to assess participants readiness for end-of-life conversations (Table 1). Items were based on qualitative research about stages in end-of-life planning, barriers and facilitators. Three domains included first, readiness to engage in end-of-life thoughts and conversations alike (readiness). Second, knowledge about personal barriers, facilitators and topics to discuss (communication experience) and third, congruence between values and life before and during palliative treatment (importance of values). All items were developed related to five stages of advance care planning behavior (Fried et al., 2010) and qualitative questions asked. Items 2 and 11 were based on findings that end-of-life conversations may be too emotional and frightening, while importance of values in life was simultaneously acknowledged to ensure they were honored at a time, when patients were not able to express themselves (Fried et al., 2009). Items 1, 3, and 10 were based on beliefs in end-of-life conversations to be normal and relevant given a certain age or prognosis (Simon et al., 2015). Additionally, discomfort of end-of-life conversations, expression of emotions and fear of death and dying were considered. Also, the importance to address former experiences with life-sustaining treatments and the understanding of importance of end-of-life conversations were discussed. Items 11 and 13 were developed based on Dignity Therapy (Chochinov et al., 2005) that uses therapeutic life review to express and remember what is important for each individual and to develop an understanding of what people would want at the end of life. Items 4 to 9 and 12 were developed based on personal therapeutic experiences and research (Fried et al., 2010). After development, three independent researchers controlled for content validity. All 13 items were presented as statements and rated on a 6-point Likert-Scale from zero (absolutely disagree) to five (absolutely agree). Items 1 to 7 were expected to be related to participants readiness to engage in end-of-life conversations. Items 2 and 3 were inversely coded. Items 8 to 10 were expected to be related to participants experience with communication and conversation regarding end-of-life, with items 11 to 13 being created in regards to the importance of values at the end of life.

**Table 1:** REOLC Scale for a community sample.

**Item**	**English**	**German**
1	I believe that dealing with the end of life is part of life.	Die Beschäftigung mit dem Lebensende gehört für mich zum Leben dazu.
2	For me, experiencing life at the present moment is way more important than talking about the end of life.	Für mich ist das Leben im Hier und Jetzt viel wichtiger als über das Lebensende zu sprechen.
3	I avoid dealing with the finite nature of my life.	Ich vermeide es, mich mit der Endlichkeit des eigenen Lebens auseinander zu setzen.
4	For me it makes sense to talk about death and dying with my family/friends.	Für mich ist es sinnvoll, mit meinen Angehörigen/Freunden über das Thema Tod und Sterben zu sprechen.
5	Dealing with the end of life allows me to experience life more intensively at the present moment.	Die Beschäftigung mit dem Lebensende lässt mich im Hier und Jetzt intensiver leben.
6	For my friends I would recommend to deal with the finite nature of life.	Einem Freund/ einer Freundin würde ich empfehlen, sich mit der Endlichkeit des eigenen Lebens auseinander zu setzen.
7	I would like to start talking about the end of my life.	Ich möchte über mein Lebensende ins Gespräch kommen.
8	I know which topics regarding the last part of my life I would like to talk about with my relatives.	Ich weiß, welche Themen ich in Bezug auf die letzte Lebensphase mit meinen Angehürigen besprechen würde.
9	I know about my personal barriers when talking about the last part of life.	Ich wei, worin für mich Hürden bei einem Gespräch über die letzte Lebensphase bestehen.
10	I know what advantages talking about the end of my life holds.	Ich kenne die Vorteile eines Gesprächs über das Lebensende.
11	I am aware of what in life is important to me.	Ich bin mir darüber im Klaren, was mir im Leben wichtig ist.
12	Dying with dignity means to end life the way one has lived it so far.	Würdevolles Sterben bedeutet, so aus dem Leben zu treten, wie man es bislang geführt hat.
13	I have already learned a lot about life.	Ich habe bereits einiges über das Leben gelernt.

##### 2.1.2.2. Additional Self-Report Measures

Demographic data was collected using a standardized questionnaire. Divergent validity was assessed with distress (Distress Thermometer) (Mehnert et al., 2006) and depressive symptoms (PHQ-9, α = 0.89) (Kroenke et al., 2001). Convergent validity was assessed with trait gratitude (GQ-6, α = 0.82) and behavior stages of talking about end-of-life with loved ones and providing a living will (McCullough et al., 2002; Fried et al., 2010) (Supplementary Material).

#### 2.1.3. Statistical Analysis

A minimum sample size of *N* = 260 participants was based on a ratio of 20:1 (Costello and Osborne, 2005), expected to provide a strong factor structure and prevent miss-specification of factors. Sum scores and average scores were generated without missing values. Participants were excluded from analysis for missing values on REOLC (*n* = 15) or met exclusion criteria (*n* = 120). Correlations were computed using all complete pairs of observations. Early termination lead to exclusion. Drop out analysis (*n* = 221) showed no significant differences in demographic variables except age [*M*_*dropout*_ = 32.74, *SD*_*dropout*_ = 17.77, *M*_*included*_ = 41.34, *SD*_*included*_ = 20.97, *t*(444.35) = -5.31, *p* < 0.001].

##### 2.1.3.1. Exploratory Factor Solution

Exploratory factor analysis (EFA) and principal factor analysis determined underlying latent variables (Fabrigar et al., 1999; Costello and Osborne, 2005). Bartlett's Test of Sphericity was used to control for homogeneity of variances (Bartlett, 1950) and Kaiser-Meyer-Olkin (KMO) criterion controlled for factor sample adequacy. Velicers Minimum-Average-Partial-test (MAP) criteria, parallel analysis and scree-test were used to define the number of factors to be extracted (Fabrigar et al., 1999; Costello and Osborne, 2005). Several factor analyses compared extraction recommendations for best model fit using the following criteria: Each factor explained at least three items, item loadings or crossloadings ≥ 0.32 excluded from further analysis (Tabachnick and Fidell, 2005). For best possible fit promax rotation was used (Fabrigar et al., 1999). Alternative models were compared for theoretical sensibility and model fit relying on indicators such as improved χ^2^-statistic, *Root-Mean-Square-Error of Approximation* (RMSEA) and *Comparative-Fit-Index* (CFI).

### 2.2. Results

#### 2.2.1. Participant Characteristics

Participants (*N* = 349) in the community sample were aged from 18 to 88. Clinical distress was low for the majority of participants and more than 70% reported to have not filled out an advance care directive at the time of data collection (Table 2). Participants reported to be in pre-contemplation for completion of living will (44.41%) and talking to family about their end-of-life wishes (42.94%), respectively.

**Table 2:** Demographic and medical information.

**Study one - community sample**			**Study two - participants affected by cancer**		
**Characteristics**	***n***	**%**	**Characteristics**	***n***	**%**
Gender			Gender		
Female	257	73.64	Female	73	86.90
Male	91	26.07	Male	11	13.10
Education			Education		
Secondary High School	50	14.33	Secondary High School	6	7.14
A-levels	147	42.12	A-level	7	8.33
University Degree	148	42.41	University Degree	49	58.33
Other	4	1.15	Other	22	26.19
Chronic Illness			Cancer Diagnosis		
Yes	81	23.21	First	46	54.76
No	260	74.50	Second	3	3.57
Psychological Illness			Third	2	2.38
Yes	22	6.30	Recurrence	11	13.10
No	325	93.12	Free of Cancer	19	22.62
Clinical Distress			Other	3	3.57
Score <5	192	55.01	Therapy Goal		
Score ≥5	152	43.55	Curative	65	77.38
Missing	5	1.43	Palliative	19	22.62
Advance Care Directive			Cancer Type		
Yes	98	28.08	Lymphoma	13	15.48
No	251	71.92	Breast Cancer	36	42.86
			Other	30	41.67
			Active Treatment		
			Yes	58	69.05
			No	26	30.95
			Psycho-oncological Support		
			Yes	41	48.81
			No	43	51.19
			Psychotherapy		
			Yes	21	25.00
			No	63	75.00

#### 2.2.2. Exploratory Factor Analysis

Bartlett's Test of Sphericity [χ^2^(78) = 1574.1, *p* < 0.001, *N* = 349] and Kaiser-Meyer-Olkin (KMO) criterion supported sample adequacy for factor analysis (*MSA* = 0.86, *range* = 0.70-0.91). Velicer's MAP and scree test suggested a one-factor solution (*map* = 0.028), parallel analysis suggested a four-factor solution (*map* = 0.050). We conducted several principal factor analyses with promax rotation for comparison. A one-factor solution resulted in exclusion of items 11 and 12 because of low factor loadings, so that the factor was explained by 11 items and explained 38% of variance. Fit indices suggested an unfavorable fit (*RMSEA* = 0.12, 90%-*CI*[0.11;0.14], *CFI* = 0.96, α = 0.86). A four-factor solution resulted in one factor only explaining two items that correlated strongly with another factor (*r* = 0.68) and therefore was disproved. Based on the correlation and theoretical background an exploratory three-factor solution was conducted. Compared to the one-factor solution, three factors [*RMSEA* = 0.08, 90%-CI[0.065;0.095], *CFI* = 0.99] explained correlations best (△χ^2^(2) = 138.35, *p* < 0.001). One factor readiness explained eight items and two factors communication and values explained three items, respectively (Table 3). Factor loadings ranged from λ = 0.35–0.73, item difficulty, inter-item correlation and item-whole correlation were good, internal consistency ranged from α = 0.57–84 (Table 3).

**Table 3:** Study one.

**Item**	**Readiness**	**Communication**	**Values**	**M**	**SD**	**Difficulty**	**Variance**	**r_***itc***_**	**r_***iic***_**	**α**
1	0.73			3.69	1.08	61.56	1.17	0.68	0.42	0.81
2	0.54			1.80	1.22	29.99	1.49	0.51	0.46	0.84
3	0.73			3.24	1.30	53.96	1.70	0.59	0.44	0.83
4	0.52			3.20	1.17	53.30	1.37	0.68	0.42	0.81
5	0.55			2.93	1.24	48.76	1.53	0.60	0.44	0.82
6	0.71			2.70	1.37	45.03	1.87	0.78	0.40	0.80
7	0.56			2.23	1.29	37.11	1.67	0.74	0.41	0.80
8		0.52		2.89	1.32	48.19	1.75	0.70	0.49	0.65
9		0.70		2.59	1.20	43.17	1.44	0.61	0.57	0.73
10		0.67		2.91	1.27	48.52	1.62	0.72	0.46	0.63
11			0.71	3.93	0.89	65.47	0.80	0.60	0.22	0.34
12			0.35	3.21	1.25	53.58	1.57	0.38	0.43	0.60
13			0.60	3.67	0.88	61.13	0.77	0.55	0.28	0.42

*Exploratory factor analysis with promax rotation in a community sample*.

*N = 349. Factor loadings, average score (M), standard deviation (SD), item difficulty and variance, item-whole correlation (r_itc_), inter-item correlation (r_iic_), and α if item was dropped for each factor respectively. Items two and three were coded reverse*.

#### 2.2.3. Validity

Convergent validity was supported by significant correlations of all sub-scales with gratitude and behavior stages for completing a living will and talking to loved ones about end-of-life. Participants with an advance care directive were more likely to report being ready (*r* = 0.25, *p* < 0.001) for EOL conversations, having communication experience (*r* = 0.33, *p* < 0.001) and see their values as important for further treatment (*r* = 0.30, *p* < 0.001). Divergent validity was supported by non-significant and low correlations of all sub-scales with depression and distress (Table 4).

**Table 4:** Study one.

	**Variable**	**M**	**SD**	**1**	**2**	**3**	**4**	**5**	**6**	**7**
1	Living will	2.63	1.62							
2	Talking about EOL	2.80	1.73	0.38^**^						
3	PHQ-9	7.72	5.67	–0.16^**^	–0.06					
4	Distress	4.24	2.24	–0.00	–0.02	0.31^**^				
5	GQ-6	28.99	2.50	–0.08	0.03	–0.06	–0.08			
6	Readiness	2.83	0.89	0.28^**^	0.29^**^	–0.04	0.05	0.14^**^		
7	Communication	2.80	1.04	0.34^**^	0.30^**^	0.03	0.07	0.09	0.57^**^	
8	Values	3.60	0.74	0.28^**^	0.19^**^	–0.10	–0.05	0.06	0.16^**^	0.33^**^

*Scale inter-correlations in a community sample*.

*N = 349. M and SD are used to represent mean and standard deviation, respectively*.

* indicates p < 0.05

** *indicates p < 0.01*.

*Readiness for End-of-Life Conversation (Readiness), communication experience (Communication), importance of values in life (Values), Distress Thermometer (DT), Patient Health Questionnaire 9 (PHQ-9), Gratitude-Questionnaire (GQ-6). Readiness to fill out an advance directive (Living Will) and readiness to talk about End-of-Life with family members (Talking about EOL) are displayed as numeric values. Low values indicate lower stages of health behavior (e.g., one = pre-contemplation), higher values indicate higher stages of health behavior (e.g., five = maintenance)*.

## 3. Study Two

Study two (Berlin et al., 2020) compared the effect of two online interventions to reduce burden of end-of-life on former and present cancer patients. The present study relies on data sets prior to intervention start and only presents relevant variables to study cause.

### 3.1. Materials and Methods

#### 3.1.1. Inclusion Criteria and Procedure

Participants were eligible when aged 18 years or older, understood German sufficiently, reported cancer diagnosis and had access to internet. Participants were excluded for suicidal thoughts or acute psychosis (Supplementary Material). Participants were recruited with flyer, email and media promotion.

#### 3.1.2. Measures

##### 3.1.2.1. Readiness for End-of-Live Conversation Scale

The factor solution found in study one was validated in a sample of cancer patients. No changes were made, but it was controlled for an underlying common factor based on exploratory factor analysis.

##### 3.1.2.2. Additional Self-Report Measures

Demographic data was collected using a standardized questionnaire. Medical and psychological data were based on self-report. Participants were asked to report the time of diagnosis, cancer site, diagnosis type, treatment status and treatment goal. Usage of psychological support and psychotherapy were assessed. Psychological distress (distress thermometer) and death anxiety (DADDS-G, α = 0.90) were used for divergent validity (Mehnert et al., 2006; Engelmann et al., 2016). Additionally, depression and general anxiety were measured using the Patient Health Questionnaire-4 (PHQ-4). It measures the two key criteria for depression (PHQ-2, α = 0.81) and generalized anxiety disorder (GAD-2, α = 0.73), respectively (Kroenke et al., 2009). Fear of progression (FOP-Q, α = 0.86) assessed dysfunctional fear related to cancer recurrence (Herschbach et al., 2005). Correlation with all questionnaires was expected to be small and non-significant. Convergent validity was established based on trait gratitude (GQ-6, α = 0.75) (McCullough et al., 2002). Correlations were expected to be moderate and significant.

#### 3.1.3. Statistical Analysis

Minimal sample size was estimated in relation to the severity of a cancer diagnosis with 5 times the number of items (*N*_*min*_ = 5x13 = 65) as sufficient. Three participants were excluded for missing data on REOLC. There were no dropouts in study two.

##### 3.1.3.1. Structural Equation Modeling

Structural equation modeling (SEM) was used to confirm factor structure. Parameters were fixed in order to standardize factor loadings and estimated using maximum likelihood (ML) method with Sattora-Bentler-correction and robust standard errors to control for violations of multivariate normality. Evaluation of the model was based on χ^2^-test, *Root-Mean-Square-Residual* method (SRMR). A combination of RMSEA (>0.06) and SRMR (<0.09) was recommended for small sample sizes (*N* ≤ 250) (Hu and Bentler, 1999). The χ^2^-test supported model fit if χ^2^/*df* <2 (Schermelleh-Engel et al., 2003). Item analysis provided factor score, item difficulty, item variance, inter-item correlation (*r*_*iic*_), and item-whole correlation (*r*_*itc*_).

### 3.2. Results

#### 3.2.1. Participant Characteristics

Demographic variables varied between included and excluded participants (Table 2). Included participants (*n* = 84, *M* = 45.25, *SD* = 13.29) differed significantly to excluded participants (*n* = 18, *M* = 32.06, *SD* = 12.53) in age [*t*(100) = 3.86, *p* < 0.001] and distress [*t*(31.76) = –3.96, *p* < 0.001]. Excluded participants reported to be more distressed (*M* = 6.89, *SD* = 1.64) than included participants (*M* = 5.08, *SD* = 2.21). There were no significant differences for all other variables (*p*>0.05). The majority of included participants reported to have been diagnosed in 2019 (25%), had been diagnosed for the first time and were diagnosed with breast cancer. Participants reported ongoing treatment (69.05%) and a palliative treatment goal (22.62%), and more use of psycho-oncological support than psychotherapy. On average, participants reported mild scores of depression and general anxiety, moderate death anxiety, fear of recurrence (54.76%) and clinical significant distress (61.90%, Table 5).

**Table 5:** Study two.

		**M**	**SD**	**1**	**2**	**3**	**4**	**5**	**6**
1	REOLC	3.16	0.85						
2	DT	5.08	2.21	0.08					
3	PHQ-2	1.44	1.15	0.15	0.48^**^				
4	GAD-2	1.85	1.33	–0.11	0.53^**^	0.61^**^			
5	FOP-Q	33.49	9.34	–0.04	0.59^**^	0.29^*^	0.33^**^		
6	DADDS-G	26.93	8.90	–0.07	0.46^**^	0.29^*^	0.41^**^	0.59^**^	
7	GQ-6	36.14	4.62	0.44^**^	0.18	–0.10	–0.03	–0.00	–0.09

*Scale inter-correlations in a population affected by cancer*.

*N = 84. M and SD are used to represent mean and standard deviation, respectively*.

* p < 0.05

** *p < 0.01*.

*REOLC, Readiness for End-of-Life Conversation Scale; DT, Distress Thermometer; PHQ-2, Patient Health Questionnaire 2; GAD-2, General Anxiety Questionnaire 2; FOP-Q, Fear of Progression Questionnaire; DADDS-G, Death And Dying Distress Scale German version; GQ-6, Gratitude Questionnaire*.

#### 3.2.2. Structural Equation Modeling

**Figure 1:**
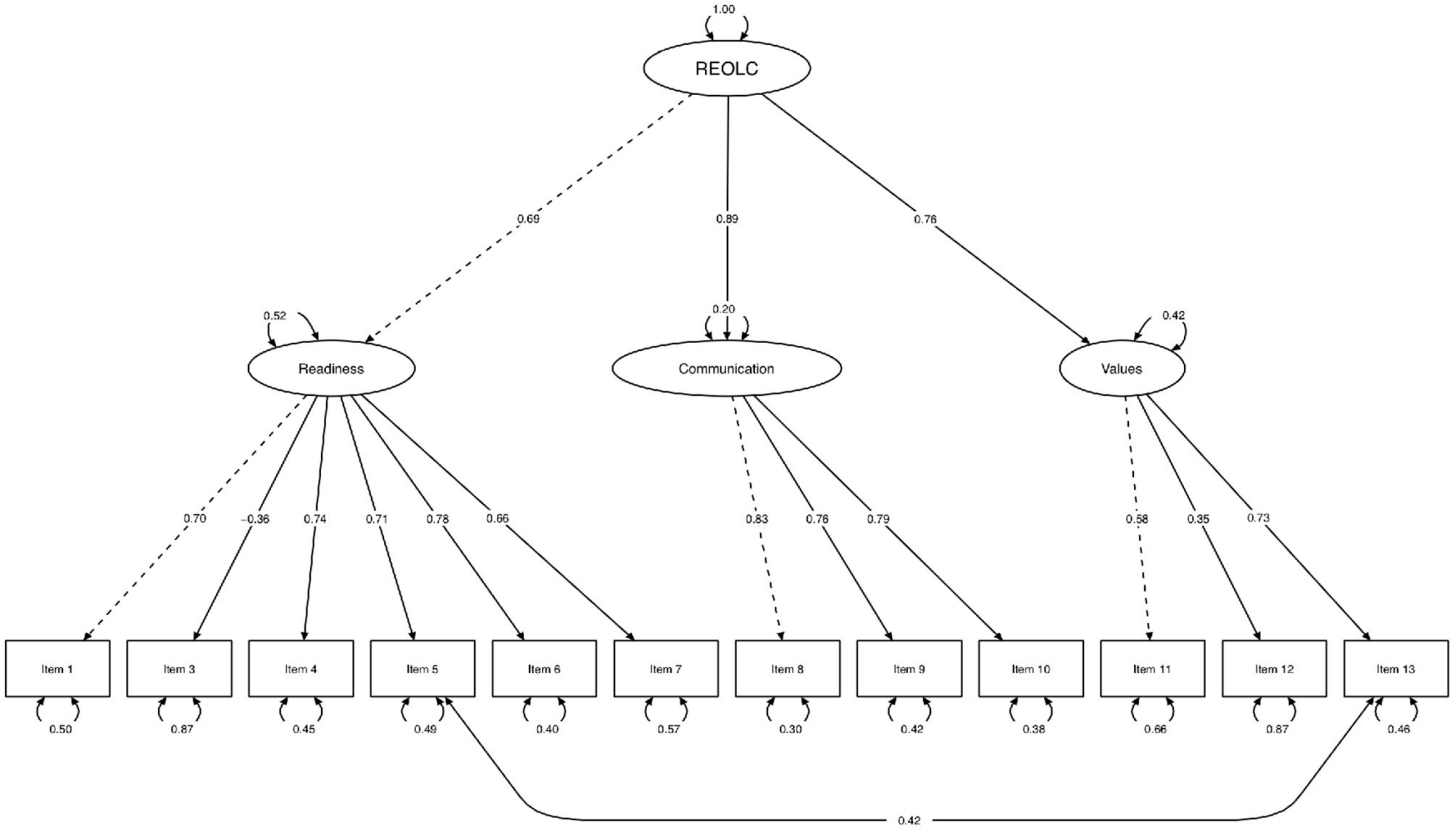
Structural equation path model. Maximum-Likelihood method, Satorra-Bentler-correction, robust standard errors and standardized parameter estimation (*N* = 84). One common latent factor readiness (REOLC) for cancer patients. Exclusion of item 2 because of low factor loadings. Variances (one-headed arrows), covariances (double-headed arrows), marked variables (dashed line), manifest variables (rectangles), latent variables (ellipses).

Bartlett's Test of Sphericity [χ^2^(78) = 402.52, *p* < 0.001, *N* = 84] and KMO-criterion supported sample adequacy (*MSA* = 0.81, *range* = 0.68-0.89). Based on findings in study one, we conducted SEM with one common latent factor of readiness. Item 2 was excluded because of low explanatory value and low factor loadings (${\text{R}}_{item2}^{2}=0.036$, λ_*item*2_ = –0.19). Correlation of item five and item 13 was high and therefore added to the alternative model (Figure 1, Table 6). Model fit was good: Corrected χ^2^-test was not significant [χ^2^(50) = 59.18, *p* > 0.05, *Satorra*−*Bentler*−*correction* = 1.27], with χ^2^/*df* = 1.184, *rRMSEA* = 0.053 (90%-*CI*[0.000;0.100]) and *rSRMR* = 0.072. Readiness explained 48.1%, Communication 79.9%, and Values 58.1% of variance.

**Table 6:** REOLC scale for a population affected by cancer.

**Item**	**English**	**German**
1	I believe that dealing with the end of life is part of life.	Die Beschäftigung mit dem Lebensende gehört für mich zum Leben dazu.
3	I avoid dealing with the finite nature of my life.	Ich vermeide es, mich mit der Endlichkeit des eigenen Lebens auseinander zu setzen.
4	For me it makes sense to talk about death and dying with my family/friends.	Für mich ist es sinnvoll, mit meinen Angehörigen/Freunden über das Thema Tod und Sterben zu sprechen.
5	Dealing with the end of life allows me to experience life more intensively at the present moment.	Die Beschäftigung mit dem Lebensende lässt mich im Hier und Jetzt intensiver leben.
6	For my friends I would recommend to deal with the finite nature of life.	Einem Freund/ einer Freundin würde ich empfehlen, sich mit der Endlichkeit des eigenen Lebens auseinander zu setzen.
7	I would like to start talking about the end of my life.	Ich möchte über mein Lebensende ins Gespräch kommen.
8	I know which topics regarding the last part of my life I would like to talk about with my relatives.	Ich weiß, welche Themen ich in Bezug auf die letzte Lebensphase mit meinen Angehärigen besprechen würde.
9	I know about my personal barriers when talking about the last part of life.	Ich weiß, worin für mich Hürden bei einem Gespräch über die letzte Lebensphase bestehen.
10	I know what advantages talking about the end of my life holds.	Ich kenne die Vorteile eines Gesprächs über das Lebensende.
11	I am aware of what in life is important to me.	Ich bin mir darüber im Klaren, was mir im Leben wichtig ist.
12	Dying with dignity means to end life the way one has lived it so far.	Würdevolle Sterben bedeutet, so aus dem Leben zu treten, wie man es bislang geführt hat.
13	I have already learned a lot about life.	Ich habe bereits einiges über das Leben gelernt.

#### 3.2.3. Item Analysis

Average item scores ranged from *M* = 2.50–3.93 (*SD* = 1.05–1.62). Items rated highest were *I have already learned a lot about life* (item 13), *I am aware of what in life is important to me* (item 11) and *I believe that dealing with the end of life is part of life* (item 1). Item difficulty (*d* = 50.00–78.57, σ = 1.10–2.61), item-whole correlations (*r*_*itc*_ = 0.31–0.76) and inter-item correlations (*r*_*iic*_ = 0.31–0.36) were acceptable. Internal consistency was good (α = 0.86, 95%-*CI*[0.81;0.90], Table 7).

**Table 7:** Study two.

	**Item**	**M**	**SD**	**Difficulty**	**Variance**	**r_***itc***_**	**r_***iic***_**	**α**
1	I believe that dealing with the end of life is part of life.	3.73	1.23	74.52	1.50	0.61	0.32	0.84
3	I avoid dealing with the finite nature of my life.	3.36	1.42	67.14	2.02	0.37	0.35	0.86
4	For me it makes sense to talk about death and dying with my family/friends.	3.04	1.34	60.71	1.79	0.69	0.31	0.84
5	Dealing with the end of life allows me to experience life more intensively at the present moment.	3.32	1.42	66.43	2.00	0.66	0.32	0.84
6	For my friends I would recommend to deal with the finite nature of life.	2.77	1.62	55.48	2.61	0.68	0.32	0.84
7	I would like to start talking about the end of my life.	2.50	1.40	50.00	1.96	0.61	0.32	0.84
8	I know which topics regarding the last part of my life I would like to talk about with my relatives.	2.80	1.42	55.95	2.02	0.71	0.31	0.84
9	I know about my personal barriers when talking about the last part of life.	2.68	1.43	53.57	2.05	0.59	0.33	0.84
10	I know what advantages talking about the end of my life holds.	2.57	1.57	51.43	2.46	0.76	0.31	0.83
11	I am aware of what in life is important to me.	3 86	1.11	77.14	1.23	0.41	0.34	0.85
12	Dying with dignity means to end life the way one has lived it so far.	3.32	1.32	66.43	1.74	0.31	0.36	0.86
13	I have already learned a lot about life.	3.93	1.05	78.57	1.10	0.58	0.33	0.85

*Descriptive and item statistics for REOLC in a population affected by cancer*.

*N = 84. Average score (M), standard deviation (SD), factor loadings, item difficulty and variance, item-whole correlation (r_itc_), inter-item correlation (r_iic_) and α if item was dropped. Item two For me, experiencing life at the present moment is way more imporant than talking about the end of life. was removed from REOLC because of weak factor loadings λ ≤ |0.32|*.

#### 3.2.4. Criterion Validity

Results showed no significant correlations with general anxiety, fear of recurrence, death anxiety, distress, and depression. Readiness correlated positive and significant with gratitude (Table 5).

## 4. Discussion

A challenge in end-of-life conversations is the moment of confrontation and prevention of emotional burden for all participants. The purpose of this study was to create and validate a questionnaire that reliably assesses readiness for end-of-life conversations in a community sample and a population affected by cancer. Study one found three underlying factors in a community sample. Participants were ambivalent to avoid (item 3) or include (item 1) end-of-life discussions in life. Contrary to acceptance of necessity and beneficence regarding end-of-life conversations, average avoidance tendencies were high. These findings are in alignment with previous research: When illness and impeding death become a reality, caregivers often experience a reduction in readiness to engage in mortality communication, avoiding confrontation by subconsciously trying to reduce psychological burden (Bachner et al., 2020). Distraction or delay in conversations may be used to prevent individuals from the experience of negative affect (Arndt et al., 2007). This emphasizes the need for repeated reminders and sensitive strategies to support individuals in their choice to address fears when talking about death and dying.

Study two controlled for a common latent factor *readiness*. For cancer patients, clear separation of death from life (item 2) was of low explanatory value and therefore excluded. In comparison to a community sample, ignoring the possibility of death is impossible for cancer patients, because it is unwillingly introduced at time of diagnosis (Ferrell et al., 1998; Baum and Andersen, 2001). Death related health behavior, however, may still be avoided (item 3), because subconscious defense mechanisms prevent the accessibility of death-thoughts when making decisions under emotional strain. Then hope and beliefs in a just world are maintained in order to minimize threats to self (Arndt et al., 1997, 2007). A direct approach by physicians may increase accessibility of death-thoughts and avoidance of end-of-life conversations. An indirect approach using a questionnaire may reduce accessibility or decrease emotional strain, subsequently increasing interest in health behavior (Arndt et al., 2007). Further, constant changes in health status may repeatedly suppress and activate thoughts about death. Patients would report a change in readiness for end-of-life conversations accordingly. Indirect routine assessments may then increase chances for timely identification by health care providers. Items with high explanatory value were related to knowledge about personal preferences in topics and advantages of end-of-life conversations (item 8 and 10). Cancer patients have broader knowledge about medical side effects and treatment options compared to the average person. Also, thoughts about end-of-life preferences possibly have been necessary based on prognosis, family history or personal experiences. Knowledge about topics and advantages may not result in increased health behavior action, but includes contemplation and possible openness toward end-of-life conversations.

Despite openness for end-of-life conversations, hesitation may result from lack of trust in physicians, avoidance of psychological burden and informational deficits (Higginson and Costantini, 2002; Zhang and Siminoff, 2003; Goldsmith et al., 2007; Stone et al., 2012; Lambert South and Elton, 2017). Additionally, oncologists may experience difficulties to anticipate the moment to start and to engage in end-of-life conversations, often waiting for patient cues. Patients, however, may be reluctant, not ready or waiting for physicians initiation. Further, families may avoid end-of-life conversations in order to protect patients and themselves (Granek et al., 2013). A comparable questionnaire in the United States of America provided insights on the discrepancy of physicians assessment. Although patients reported to be ready, 83% of physicians did not initiate end-of-life conversations, 44.7% felt their patients were not ready and 13% were utterly surprised by self-reports (Kogan and Taguchi, 2020). An indirect assessment of readiness offers information without activation of resistance and provides a neutral tool for patients to voice and practitioners to identify readiness. Strategies and information to overcome barriers in the way of best medical and psychological care can be provided accordingly.

For both studies, readiness was associated with gratitude. Research found that focusing on life with gratitude and evaluating past events gratefully reduced death anxiety and increased likelihood of health behavior engagement (Lau and Cheng, 2011). Results of the present study indicate that high levels of gratitude lead to a broader understanding of positive outcomes associated with end-of-life conversations and thereby increase readiness to engage in such. Also, neither distress nor any other measure of psychological burden was associated with readiness for end-of-life conversations. Readiness for end-of-life conversations is an unrelated construct that assesses the openness to engage in a behavior despite high distress, highlighting the importance of an additional measurement in cancer care.

Findings of the present study underlie the following statistical limitations: First, different measures for convergent and discriminant validity were used in both studies, reducing comparability. However, measurements taken were still viable measures to yield empirical justification for discriminant validity in general. Second, sample size in study two did not meet requirements for structural equation modeling. Therefore, statistical corrections were performed to ensure adequate interpretation of the results. Third, uptake of health behavior after participation was not assessed. Scores of readiness could not be compared with behavior intention and action. Fourth, selection of participants may bias our results. Participants may have generally been open to and interested in the subject of end-of-life. Finally, due to item modification in study two we additionally recommend further validation using comparable samples of cancer patients. Overall, development and validation were based on two highly selective and small study samples with different health status and relation to end-of-life. Future studies should include larger samples and compare factor structure across varying conditions. Based on the present findings, generalizabilty of factor structure is not given and should be considered when questionnaire is used.

For clinical implications, sample size in the population affected by cancer was too small to make further assumptions about differences in factor structure and readiness for end-of-life conversations based on health status. In addition to larger sample sizes, future studies should use medical information provided by clinicians to make assumptions about differences in readiness across cancer site, active treatment or treatment goal. Readiness may vary dependent on treatment and expected treatment outcome. For curative patients, readiness at the time of diagnosis may not be high but change over the course of treatment, experiences with negative side effects, personal loss and changes in health status. For palliative patients, end-of-life conversations are important to provide patient-centered advanced care and decrease burden. Routine assessment of readiness would enable clinicians to identify changes in readiness and to provide information, guidance and support accordingly. In Germany, the S3-guidelines for palliative care (Leitlinienprogramm Onkologie, 2020) preset criteria for patient-centered care and shared decision making processes. They include incorporation of patient and family needs in addition to relief of strain, assessment of need for information, hopes and fears regarding treatment and knowledge before additional information is presented. They further highlight the importance of sensible conversations about death and dying, medical decisions at the end of life and early and repeated options to discuss end-of-life plans. Patients and families are expected to be included in the decision making process and offered guidance from health professionals. The REOLC scale may be used to identify overall readiness, but also to focus on individual barriers that may prevent patients to address the topic of end-of-life care first. It further may ease the initiation of end-of-life conversations for clinicians, patients and families alike. However, at present additional research is needed to explicitly validate the REOLC scale for palliative cancer patients.

While it has been proven beneficial for palliative patients to engage in advance care planning (Bischoff et al., 2013), research of the effect on former cancer patients or curative patients is scarce. However, assessing readiness for end-of-life conversations and different time points in cancer care should not be undervalued. For one, difficulties to unalterably interpret treatment as palliative, possible changes from curative to palliative treatment because of complications, changes in possibilities of a cure and advances in cancer stage add to the uncertainty of diagnosis not only for patients, but also for physicians (Leitlinienprogramm Onkologie, 2020). Also, readiness for end-of-life conversations may be higher if the death threat is not imminent, but still relevant because of diagnosis with cancer (Arndt et al., 2007). Until today, the S3-guidelines recommend questionnaires and screening methods for a variety of burden (Leitlinienprogramm Onkologie, 2020), but no tool to assess readiness for end-of-life conversations. The REOLC scale fills this gap in palliative care and offers a possibility to support health care practitioners by indicating readiness for end-of-life conversations in patients and family members alike. By application of the questionnaire independently of health status, practitioners gain insights in readiness of not only palliative patients, but also curative patients and are able to track and act accordingly over the course of diagnosis, treatment and follow ups.

One possibility to successfully screen for readiness would be the identification of a clinical cut-off, readiness scores in relation to health behavior stages and the minimal amount of readiness needed to start conversations and interventions (Westley and Briggs, 2004; Fried et al., 2009, 2010). Based on cut-off criteria and health behavior stages, interventions can address and modify specific barriers and support facilitators of end-of-life conversations. Additional qualitative analyses including patients and care-givers perspectives may provide insights and highlight mechanisms that can be targeted specifically. The REOLC scale enables researchers to evaluate such interventions for a community sample and cancer patients, respectively von Blanckenburg et al. (2020). With the REOLC Scale we may be one step closer to develop strategies that enable cancer patients and caregivers to change between stages more easily.

## 5. Conclusion

Conversations about end-of-life are referred to as necessary and beneficial. Readiness for these conversations, however, varies and therefore needs to be assessed before interventions or conversations are issued. A questionnaire to assess readiness is the Readiness for End-of-Life Conversations (REOLC) Scale of the present study. For a community sample, 13 items cover readiness, communication experience and importance of values thereby acknowledging the paradox of simultaneous desire to engage and avoid end-of-life conversations. For a population of cancer patients, a one-factor model with 12 items was suggested. The factor *readiness* acknowledges the difficulty for cancer patients to avoid the topic of death and end-of-life preparations. Model fit, convergent and discriminant validity were good. Future studies should validate the questionnaire in larger populations and different settings and assess changes in readiness and health behavior intentions.

## Data Availability Statement

The datasets presented in this article are not readily available because: Data will only be shared at request after data collection of accompanying studies is completed. Requests to access the datasets should be directed to pia.berlin@uni-marburg.de.

## Ethics Statement

The studies involving human participants were reviewed and approved by Ethics Committee of the department of psychology at Philipps-University of Marburg. The patients/participants provided their written informed consent to participate in this study.

## Author Contributions

PB, PvB, WR, NL, and KN contributed to study design. PvB, WR, NL, and KN designed the questionnaire. PB and PvB analyzed and interpreted the data. PB and PvB drafted the manuscript are responsible for the content of manuscript. NL, KN, CS, and WR provided critical feedback and revisions. All authors approved the final draft for submission.

## Conflict of Interest

The authors declare that the research was conducted in the absence of any commercial or financial relationships that could be construed as a potential conflict of interest.
