# Supplementary material for: Development and Validation of the Readiness for End-of-Life Conversations (REOLC) Scale
Source: Front Psychol. 2021 Mar 19;12:662654. doi: 10.3389/fpsyg.2021.662654 (PMC8044973; doi:10.3389/fpsyg.2021.662654)
Supplement: Supplementary file 1 [file Data_Sheet_1.PDF]

Supplementary Material

1 STUDY ONE

1.1 Materials and Methods

1.1.1 Exclusion Criteria

Exclusion criteria were assessed using the Beck-Depression Inventory II (BDI-II) and Brief Symptom Inventory (BSI). One item of the BDI-II (*During the past two weeks including today, did you have any suicidal thoughts?*) (Beck et al., 1996) was answered on a 4-point Likert Scale from zero (*I do not think about harming myself*) to three (*I would kill myself if I had the chance to*). Participants were excluded from study when scores were equal to or higher than two. Items (e.g. perception of voices or conversations nobody else could hear, the perception of phantoms) of the BSI were answered on a dichotomous scale (yes/no) (Wittchen et al., 1997). Participants who agreed to more than three items were excluded prior to data analysis.

1.1.2 Measures

Distress (Distress Thermometer) over the past week was assessed on a visual scale from zero to 10 (Mehnert et al., 2006). Clinical cut-off is internationally recommended at values greater than four. Depressive symptoms were rated on a 4-point Likert-Scale ranging from zero (never) to three (always). Trait gratitude (GQ-6) was assessed on six items and scores were rated on a 7-point Likert-Scale from one (strongly disagree) to seven (strongly agree), higher values indicating higher gratitude (McCullough et al., 2002). Behavior stages were categorized into five stages: Pre-contemplation, contemplation, preparation, action and maintenance. Higher scores indicated a higher stage (Fried et al., 2010).

2 STUDY TWO

2.1 Materials and Methods

2.1.1 Exclusion Criteria

Exclusion criteria were assessed using the BDI-II (see study one) and BSI. In study two, the five items of BSI were rated from zero to three. Cut-off was based on normative ratings for acute psychosis at BSI $\geq$ 70.

2.1.2 Measures

Death anxiety was assessed with the Death and Dying Distress Scale (DADDS-G) (Engelmann et al., 2016). Nine items were rated from zero (*I was not distressed by this thought or concern*) to four (*I experienced extreme distress*). Higher values indicate severe distress. The Patient Health Questionnaire-4 (PHQ-4) is successfully used as screening instrument for panic, social anxiety and post-traumatic stress disorders. Items are rated on a 4-point Likert-Scale from zero (not at all) to three (nearly every day) with a total score ranging from zero to 12. For both criteria cut-offs separate between normal (zero to two), mild (three to five), moderate (six to eight) and severe (nine to 12) depression or general anxiety (Kroenke et al., 2009). Fear of recurrence (FOP-Q) was rated on a 5-point Likert-Scale from one (never) to five ("very often"). Total scores range from 12 to 60, with a cut off at FOP-Q $\geq$ 34 (Herschbach et al., 2005).

REFERENCES

Beck, A. T., Steer, R. A., and Brown, G. (1996). *Manual for the Beck Depression Inventory–II* (San Antonio, TX: Psychological Corporation)

Engelmann, D., Scheffold, K., Friedrich, M., Hartung, T., Schulz-Kindermann, F., Lordick, F., et al. (2016). Death-related anxiety in patients with advanced cancer: Validation of the german version of the death and dying distress scale (DADDS-G). *Journal of Pain and Symptom Management* 52, 582–587. doi:10.1016/j.jpainsymman.2016.07.002

Fried, T. R., Redding, C. A., Robbins, M. L., Paiva, A., O'Leary, J. R., and Iannone, L. (2010). Stages of change for the component behaviors of advance care planning. *Journal of the American Geriatrics Society* 58, 2329–2336. doi:10.1111/j.1532-5415.2010.03184.x

Herschbach, P., Berg, P., Dankert, A., Duran, G., Engst-Hastreiter, U., Waadt, S., et al. (2005). Fear of progression in chronic diseases: psychometric properties of the fear of progression questionnaire. *Journal of Psychosomatic Research* 58, 505–511. doi:10.1016/j.jpsychores.2005.02.007

Kroenke, K., Spitzer, R. L., Williams, J. B., and Löwe, B. (2009). An ultra-brief screening scale for anxiety and depression: the phq–4. *Psychosomatics* 50, 613–621. doi:10.1016/S0033-3182(09)70864-3

McCullough, M. E., Emmons, R. A., and Tsang, J.-A. (2002). The grateful disposition: A conceptual and empirical topography. *Journal of Personality and Social Psychology* 82, 112–127. doi:10.1037/ /0022-3514.82.1.112

Mehnert, A., Müller, D., Lehmann, C., and Koch, U. (2006). Die deutsche version des nccn distress-thermometers: empirische prüfung eines sreening-instruments zur erfassung psychosozialer belastung bei krebspatienten. *Zeitschrift für Psychiatrie, Psychologie und Psychotherapie* 54, 213–223. doi:10. 1024/1661-4747.54.3.213

Wittchen, H., Wunderlich, U., Gruschwitz, S., and Zaudig, M. (1997). *SCID: Structured Clinical Interview for DSM-IV Axis I Disorders* (Göttingen: Hogrefe)
